# Supplementary material for: Environmental influences on the Indo–Pacific octocoral Isis hippuris Linnaeus 1758 (Alcyonacea: Isididae): genetic fixation or phenotypic plasticity?
Source: PeerJ. 2015 Aug 20;3:e1128. doi: 10.7717/peerj.1128 (PMC4548502; doi:10.7717/peerj.1128)
Supplement: Supplemental Information 1 — Supplementary Material. [file peerj-03-1128-s001.doc]

**Supplementary Material**

**Environmental influences on the Indo-Pacific octocoral *Isis hippuris* Linnaeus 1758: genetic fixation or capacity for plasticity?**

Sonia J. Rowley • Xavier Pochon • Les Watling

**Supplementary material contents:**

Systematic Summary: *Isis* Linnaeus 1758 (p. 2-7)

Supplementary Figure S1 (p. 3)

Supplementary Table S2 (p. 8)

Supplementary Figure S3 (p. 9)

Supplementary Table S4 (p. 10)

Supplementary Table S5 (p. 11)

Supplementary References (p. 12-13)

Systematic Summary: *Isis* Linnaeus 1758

Sub-Class OCTOCORALLIA

Order ALCYONACEA Lamouroux 1812

Sub-Order CALCAXONIA Grasshoff 1999

Family ISIDIDAE Lamouroux 1812

Sub-Family ISIDINAE Lamouroux 1812

*Isis hippuris* Linnaeus 1758

(Figure S1)

See Bayer & Stefani, 1987 for list of references [p. 55]

*Type Material* – Unfound, however ‘authentic’ specimens were collected and fully defined from Amboina, Indonesia (Milne-Edwards & Haime, 1857).

*Diagnosis* – These arborescent colonies can be up to 1 m tall, planar or bushy with lateral or partially dichotomous branching but rarely anastomosing (net-like). Branch formations may also give a candelabrum appearance. The axis consists of alternating calcareous internodes that reduces to a fine rod through the non-scleritic and convex, dark proteinaceous (gorgonin) nodes, the former typically longer than the latter. Branching is internodal in single or multiple planes, the latter giving rise to the bushy appearance. Branch lengths and diameters are variable, and up to three short branches can arise per internode with some so close they appear nodal in highly branched colonies. The expanded calcareous cup-shaped base obliterates any trace of nodal composition particularly in older colonies. Calcareous internodes are sclerobastic (consisting of fused sclerites; but also see Nutting, 1910) with fibres arranged radially from a central core, akin to that of the sclerites bearing close resemblance to the Scleraxonia (Bayer, 1955). Internodes possess longitudinal grooves corresponding with 8 – 12 sinuous water vascular canals of ~1 mm diameter. Polyps distributed all around the branches are 0.5 – 1.25 mm apart and fully retractile to ~1.25 mm deep (and wide) into the thick coenenchyme. Such polyps bear eight lanceolate pinnate tentacles, and can possess up to three large (max. 1 mm diameter) round eggs, likely explaining the often swollen appearance of the branch tips as opposed to being a diagnostic feature (but see Nutting, 1910; Mai-Bao-Thu & Domante, 1971).

| **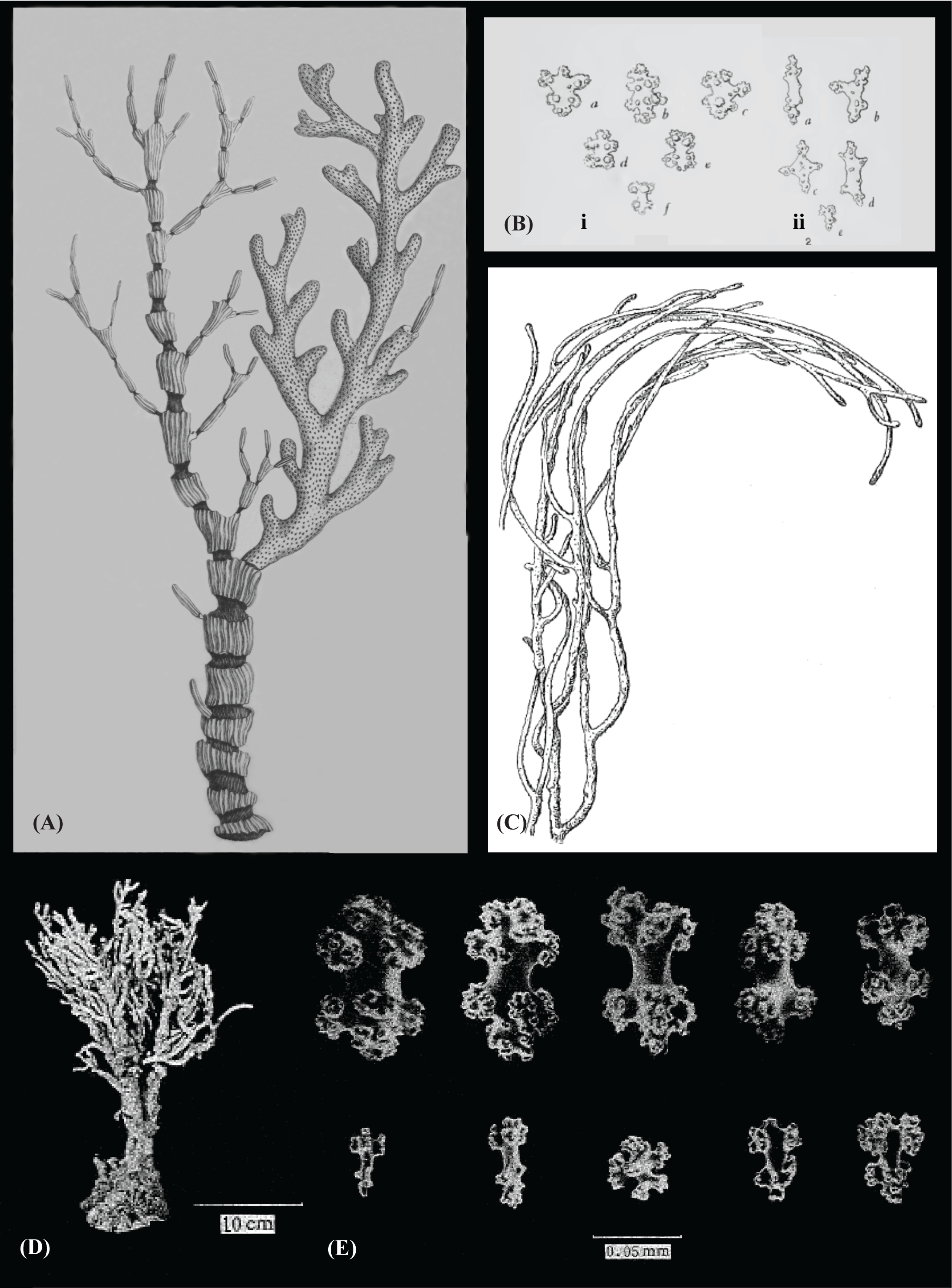** |
| --- |
| **Figure S1.** *Isis* Linnaeus, 1758 comparisons of (A) *Isis hippuris* Linnaeus, 1758 colony in Ellis & Solander, 1786; (B) sclerites of: i. *I. hippuris* and ii. *Isis reticulata* in Nutting 1910; (C) *I. reticulata* in Nutting, 1910; (D) *Isis minorbrachyblasta* Zou et al., 1991 colony and (E) sclerites. Note, images sourced from each citation respectively. |

A diverse range of sclerites exists within the thick coenenchyme (Figure S3B, E). The surface layer consists of small warty clubs 0.08 x 0.001 mm [note: typographical error pg. 55, 2^nd^ paragraph, Bayer & Stefani, 1987] typically bearing three large warts below the head wart (Figure S3B.i). Throughout the sub-surface layer some or all of the following are present in varying dimensions, asymmetry and commonly girdled: 6-, 7-, or 8-radiate capstans up to 0.19 mm in length, dumbbells/double heads (considered derivatives of 6-radiates; Bayer & Stefani, 1987) up to 0.32 mm long, warty or tuberculate spindles up to 0.25 mm long, and crosses. Very small e.g. rods of 0.07 x 0.01 mm (Bayer & Stefani, 1987), or no sclerites may be present within the polyp structures (Kölliker 1865). However smaller forms from those found within the coenenchyme (Simpson, 1906; Thomson & Simpson, 1909; Kükenthal, 1919; 1924) as well as small warty clubs with short handles ~0.055 x 0.045 mm located within the tentacles have been reported (Simpson, 1906; Thomson & Simpson, 1909).

Sclerites colourless and colonies typically light brown to mustard yellow, with slightly darker polyps.

*Distribution* – Central Indo-Pacific including Great Barrier Reef, Vanuatu, Papua New Guinea, Indonesia, Malaysia, Andaman, Philippines, Taiwan, Palau, South China Sea, Japan including Okinawa and the Ryukyu Islands.

*Remarks* – As evident from the description above, substantial phenotypic plasticity, from colony and branching structure to sclerite composition, exists in described specimens of *I. hippuris* (Wright & Studer, 1889; Simpson, 1906; Thomson & Simpson, 1909; Bayer & Stefani, 1987; Fabricius & Alderslade, 2001). Whether such plasticity is a consequence of environmental influence within and between its distributions, or significantly structured to be more than one species is unclear. Thus, for such a ubiquitous and well-known species it “has been very imperfectly described” (Thomson & Simpson, 1909) leading to “a slender basis on which to raise a superstructure of classification” (Wright & Studer, 1889). Clearly a revision of the *Isis* genus is required including thorough analyses of specimens throughout its geographic range, as such character trait variability may be ecologically dependent (Bayer & Stefani, 1987). Attempts, however, have been made to differentiate phenotypic patterns within the *Isis* genus that, even though somewhat tenuous, may equate to the morphotypes found within the WMNP (Rowley, 2014). *Isis reticulata* Nutting, 1910 and *Isis minorbrachyblasta* Zou, Huang & Wang, 1991 are therefore summarized below highlighting differences between the selected taxa.

*Isis reticulata* Nutting 1910

(Figure S1B)

See Mai-Bao-Thu & Domantay (1971) for list of references [p. 28]

*Type Material* – Syntypes: several fragments of varying sizes, ZMA COEL. no. 2721, Siboga Expedition, station 149 or 273 at Pulu Jedan, Aru Islands, Maluku, Indonesia, 13 meters on sand and shells. Fragment donated to State University of Iowa (van Soest, 1979). Specimens not located on request.

*Diagnosis* – Slender colonies, typically arborescent with long slim terminal branches that are not swollen at the ends. Few very small polyps irregularly distributed around the branches, the latter occasionally anastomosing. Sclerites of the coenenchyme bear sharp rough warts symmetrically distributed around delicate spindles and clubs the latter 0.04 – 0.06 mm in length. Some spindles curved possessing large tubercules. Irregular radiates 0.06 x 0.03 mm to 0.2 x 0.1 mm in length and width respectively, smooth warty rods 0.1 - 0.15 mm long and occasional crosses present. No polyp sclerites reported.

Sclerites colourless, colony reddish brown with slightly darker polyps in alcohol. Also noted as “brownish white” by Mai-Bao-Thu & Domantay (1971).

*Distribution* – *I. reticulata* has been documented in Indonesia (fragments from a single location 13 m depth), the Philippines (2 specimens and some fragments from a single location 12 - 15 m depth; Mai-Bao-Thu & Domantay, 1971), and Xisha Islands of China (single specimen and location; Zou, et al., 1991).

*Remarks* – *I. reticulata* is thus differentiated from *I. hippuris* on the basis of planar *versus* bushy colonies, long thin sinuous branches without swollen ends *versus* short thick antler-like branches with swollen ends, and all sclerites of a smaller size with sharp rough warts in *I. reticulata*. Sclerite differences between *I. hippuris* and *I. reticulata* have been considered questionable owing to the huge diversity in form (Stiasny, 1940; Bayer & Stefani, 1987). However, Nutting (1910) observed smaller and more sharply warted sclerites further corroborated by Kükenthal (1924) and Mai-Bao-Thu & Domantay (1971), with illustrations showing marked asymmetry (Figure S1B.ii) contrary to that described. Curiously, Nutting (1910) noted *I. reticulata* having flaccid polyps if preserved when extended due to their lack of sclerites. However, in *I. hippuris,* polyp sclerites were “not being evident on account of their small size” (Nutting, 1910) with no further discussion, lending question to their presence at all (Simpson, 1906; Simpson & Thomson, 1909; Fabricius & Alderslade, 2001; but see Bayer & Stefani, 1987; Kölliker, 1865). Conflicting sclerite images between Nutting (1910) and Mai-Bao-Thu & Domantay (1971), in addition to regional differences between specimens of *I. hippuris* (see Bayer & Stefani, 1987) having some adherence to *I. reticulata*, lends further question to its validity as a taxon. Finally, colony and polyp colouration may be an artifact of preservation; Nutting’s ‘pink’ likely from buffered formalin used at that time (note: SJ Rowley, examined a ‘pink’ specimen from the Siboga expedition at the British Natural History Museum [BNHM. 1889.6.28.18], which adhered closely to the *I. hippuris* description above and not the proposed *I. reticulata*), and Mai-Bao-Thu and Domantay’s ‘white’ from endosymbiotic bleaching not uncommon with, in particular, damaged *Isis* specimens (e.g., Thomson & Simpson, 1909). In summary, the distinction between *I. hippuris* and *I. reticulata* is conflicting and unclear, requiring further investigation.

*Isis minorbrachyblasta* Zou, Huang & Wang 1991

(Figure S1C)

*Type Material* – Holotype (G85-001) and paratype (G87-031) from two locations of the Nansha Islands, China.

*Diagnosis* – Colonies bushy with distal branches densely aggregated, themselves bearing tufts of branchlets no longer than 5 cm (ave. 3.5 cm). The short, fine branches arise from the scleritic internodes. Tiny polyps are equally distributed around the branches. Coenenchyme sclerites up to 0.140 x 0.091 mm being predominantly dumbbells and double heads with tubercules generally symmetrically arranged. Assortment of small clubs also present 0.06 x 0.025 mm with occasional crosses.

Sclerites colourless, colonies light brown in alcohol.

*Distribution* – Nansha Islands, China.

*Remarks* – Zou et al., (1991) state that the bushy non-planar colonies of *I. minorbrachyblasta* differ from the planar ones of *I. hippuris* and *I. reticulata,* in direct contrast to previous reports (e.g., Nutting, 1910; Mai-Bao-Thu & Domantay, 1971). Furthermore, the branches of *I. minorbrachyblasta* are fine, short and densely packed, whereby *I. hippuris* and *I. reticulata* are thick, short, dense, and fine, long, anastomosing and loosely packed respectively; thus *I. minorbrachyblasta* an intermediate between the two. Statistical significance between select morphological traits (branchlet and sclerite length and width) revealed differences among taxa were between *I. minorbrachyblasta* and *I. reticulata*. However, it is unclear what sclerites were used for comparative analyses, and n = 1 in all cases. Based on the information presented here, any appreciable difference in colony, branch and sclerite composition especially between *I. minorbrachyblasta* and *I. hippuris* (e.g. Nutting, 1910; Bayer & Stefani, 1987) is nebulous. Finally, Zou et al., (1991) propose *I. minorbrachyblasta* based on one or two specimens per taxon, somewhat unsatisfactory given both the nature of *Isis* phenotypic variability and analyses taken from a single region.

**Table S2.** *Isis hippuris* morphological traits summary table. All values expressed as metric or counts (± SE). Asterisk (*) indicate significantly (< *P* 0.05) informative traits selected for multivariate analyses. † Depicts low sample size.

| **Morphological Trait** | | | | **Measures/Counts** | | **Dimensions (± SE)** | | **Morphological Trait** | | **Measures/Counts** | **Dimensions (± SE)** | |
| --- | --- | --- | --- | --- | --- | --- | --- | --- | --- | --- | --- | --- |
|  |  |  |  |  |  | **Ridge 1** | **Sampela** |  |  |  | **Ridge 1** | **Sampela** |
| **Macromorphology (cm)** | | | |  | |  |  | **Micromorphology (mm)** | |  |  |  |
| ***Colony*** | | | |  | |  |  | ***Polyp*** | |  |  |  |
| *H | Colony Height | | | 48 | | 49.74 ± 3.99 | 58.78 ± 3.10 | *PD | Polyp Density | 31,761 | 88.23 ± 2.6 | 100.21 ± 5.32 |
| *W | Colony Mean Width | | | 48 | | 39.65 ± 2.24 | 58.03 ± 2.94 | Pd | Polyp Depth | 398 | 0.08 ± 0.001 | 0.08 ± 0.002 |
| **w1* | *width 1* | | | 144 | | 40.53 ± 3.24 | 65.31 ± 5.35 | pD | Polyp Diameter | 1920 | 0.04 ± 0.002 | 0.03 ± 0.001 |
| **w2* | *width 2* | | | 144 | | 57.02 ± 3.06 | 76.74 ± 3.6 | ID | Inter-polyp Distance | 1920 | 0.05 ± 0.002 | 0.05 ± 0.001 |
| **w3* | *width 3* | | | 144 | | 22.0 ± 1.35 | 31.86 ± 1.93 | Cd | Canal diameter | 1,920 | 0.02 ± 0.001 | 0.02 ± 0.002 |
| *CS | Colony overhead Spread | | | 48 | | 45.5 ± 5.10 | 78.86 ± 3.10 | C# | Canal# | 1,677 | 8.17 ± 0.14 | 8.33 ± 0.21 |
| **cs1* | *colony spread 1* | | | 48 | | 52.06 ± 5.34 | 78.86 ± 3.92 |  | |  |  |  |
| **cs2* | *colony spread 2* | | | 48 | | 38.94 ± 4.85 | 62.12 ± 3.99 | ***Sclerites*** | |  |  |  |
| ^†^B | Colony Base Width | | | 18 | | 3.47 ± 0.40 | 5.43 ± 0.64 | *CL1 | Club Length 1 | 960 | 0.073 ± 0.001 | 0.068 ± 0.001 |
| *M | Colony Mid Branch Width | | | 48 | | 1.10 ± 0.08 | 1.53 ± 0.48 | *CW1 | Club Mean Width 1 | 48 | 0.021 ± 0.000 | 0.020 ± 0.000 |
| *TW | Colony Tip Branch Width | | | 960 | | 0.63 ± 0.02 | 0.95 ± 0.05 | *c1w1* | *c1width 1* | 960 | 0.022 ± 0.001 | 0.021 ± 0.001 |
|  |  | | |  | |  |  | *c1w2* | *c1width 2* | 960 | 0.012 ± 0.000 | 0.011 ± 0.001 |
| ***sub Colony*** | | | |  | |  |  | *c1w3* | *c1width 3* | 960 | 0.030 ± 0.002 | 0.028 ± 0.001 |
| *sH | | sHeight | | 48 | | 10.8 ± 0.33 | 11.55 ± 0.24 | *CL2 | Club Length 2 | 960 | 0.072 ± 0.000 | 0.068 ± 0.002 |
| *sW | | sMean Width | 48 | | 3.15 ± 0.21 | | 2.66 ± 0.17 | *CW2 | Club Mean Width 2 | 48 | 0.033 ± 0.000 | 0.031 ± 0.001 |
| *sw1* | | *swidth 1* | | 144 | | 2.50 ± 0.14 | 2.46 ± 0.15 | *c2w1* | *c2width 1* | 960 | 0.032 ± 0.001 | 0.030 ± 0.001 |
| *sw2* | | *swidth 2* | | 144 | | 4.18 ± 0.28 | 3.37 ± 0.22 | *c2w2* | *c2width 2* | 960 | 0.016 ± 0.000 | 0.016 ± 0.001 |
| *sw3* | | *swidth 3* | | 144 | | 2.76 ± 0.32 | 1.94 ± 0.23 | *c2w3* | *c2width 3* | 960 | 0.051 ± 0.001 | 0.047 ± 0.002 |
| sML | | sMean Mother Length | | 48 | | 8.41 ± 0.33 | 9.77 ± 0.35 | *CaL | Capstan Length | 960 | 0.114 ± 0.004 | 0.101 ± 0.002 |
| *sMW | | sMean Mother Width | | 48 | | 0.44 ± 0.02 | 0.55 ± 0.02 | *CaW | Capstan Mean Width | 48 | 0.075 ± 0.001 | 0.066 ± 0.001 |
| *mw1* | | *mwidth 1* | | 144 | | 0.51 ± 0.01 | 0.62 ± 0.03 | **caw1* | *cawidth 1* | 960 | 0.086 ± 0.004 | 0.076 ± 0.003 |
| *mw2* | | *mwidth 2* | | 144 | | 0.48 ± 0.01 | 0.57 ± 0.02 | **caw2* | *cawidth 2* | 960 | 0.041 ± 0.003 | 0.036 ± 0.020 |
| *mw3* | | *mwidth 3* | | 144 | | 0.42 ± 0.01 | 0.54 ± 0.02 | **caw3* | *cawidth 3* | 960 | 0.090 ± 0.003 | 0.080 ± 0.002 |
| *sDL | | sMean Daughter Branch Length | | 48 | | 3.08 ± 0.21 | 4.44 ± 0.18 | SL | Spindle Length | 960 | 0.164 ± 0.003 | 0.162 ± 0.003 |
| *sDW | | sMean Daughter Branch Width | | 48 | | 0.42 ± 0.01 | 0.48 ± 0.01 | *SW | Spindle Mean Width | 48 | 0.069 ± 0.001 | 0.065 ± 0.001 |
| *dw1* | | *dwidth 1* | | 480 | | 0.44 ± 0.01 | 0.51 ± 0.01 | *sw1* | *swidth 1* | 960 | 0.038 ± 0.002 | 0.037 ± 0.002 |
| *dw2* | | *dwidth 2* | | 480 | | 0.41 ± 0.01 | 0.49 ± 0.01 | *sw2* | *swidth 2* | 960 | 0.080 ± 0.003 | 0.076 ± 0.002 |
| *dw3* | | *dwidth 3* | | 480 | | 0.38 ± 0.01 | 0.45 ± 0.01 | **sw3* | *swidth 3* | 960 | 0.081 ± 0.003 | 0.077 ± 0.002 |
| *MBW | | Mean Branch Width | | 48 | | 0.43 ± 0.01 | 0.52 ± 0.01 |  |  |  |  |  |
| *sTB# | | sTotal Branch# | | 48 | | 13.5 ± 1.10 | 10.83 ± 1.42 | **Total** | | **59,328** |  |  |
| sTBL | | sTotal Branch Length | | 48 | | 51.86 ± 3.49 | 52.57 ± 3.76 |  |  |  |  |  |
| *PA | | Projected sub-colony Area | | 48 | | 34.28 ± 2.65 | 30.79 ± 2.03 |  |  |  |  |  |
| *PBA | | Projected Branch Area | | 48 | | 22.41 ± 1.69 | 26.96 ± 2.06 |  |  |  |  |  |
| *Po | | Porosity | | 48 | | 1.61 ± 0.10 | 1.19 ± 0.06 |  |  |  |  |  |
|  | |  | |  | |  |  |  | |  |  |  |

| 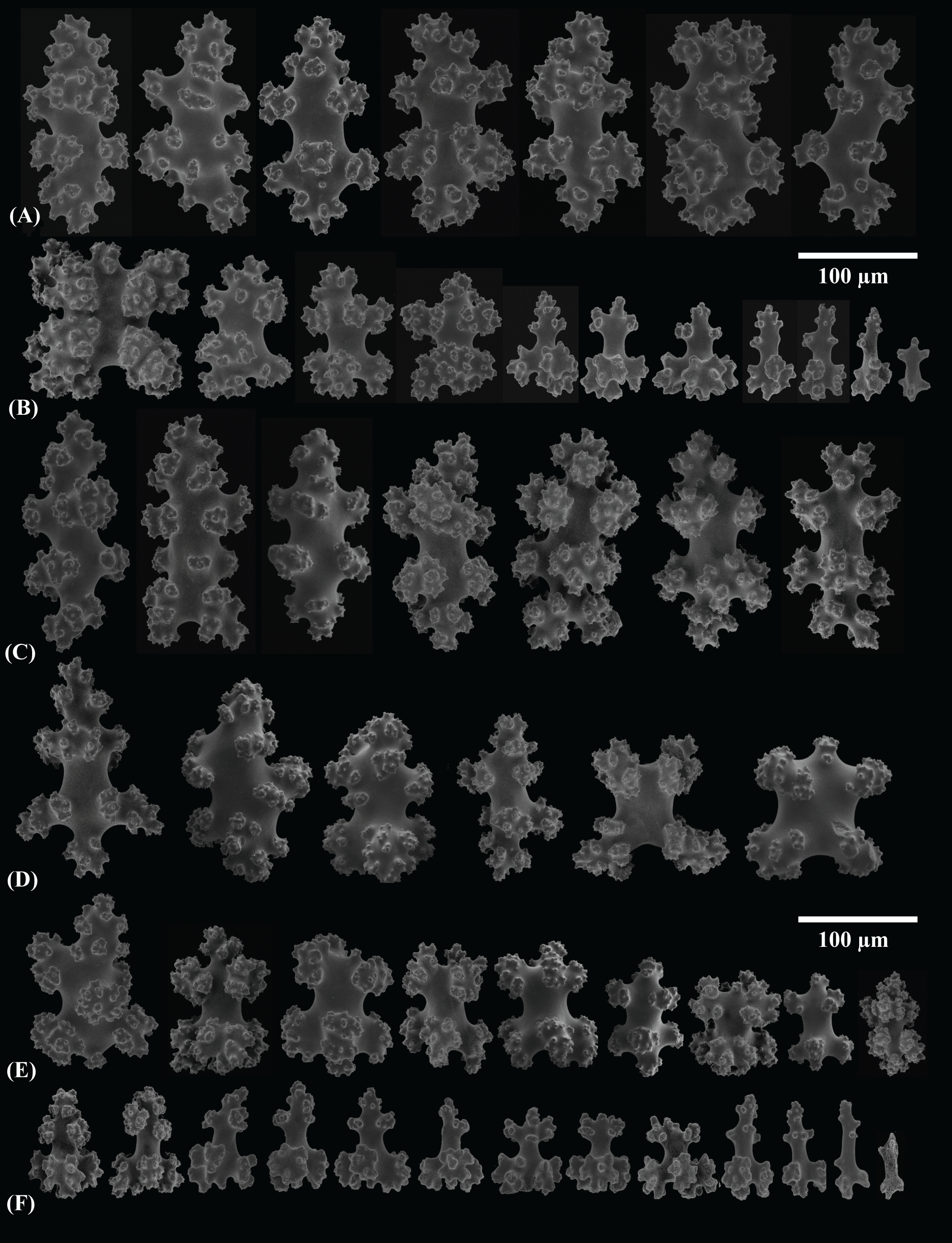 |
| --- |
| **Figure S3.** Scanning electron micrographs showing sclerite diversity of *Isis hippuris* from (A - B) Ridge 1 and (C - F) Sampela within the WMNP. Inner coenenchyme spindles (A, C, D), surface capstans and clubs (B, E, F). Small rods at the end of both (A & F). |

**Table S4.** ITS2 Accessions of octocoral outgroups used in the analyses.

| **Taxon** | **GenBank** | **Reference** |
| --- | --- | --- |
|
| [Group: Alcyoniinans] |  |  |
| Family: Alcyoniidae Lamouroux, 1812 |  |  |
| *Alcyonium digitatum* Linnaeus, 1758 | AF262347 | McFadden et al., 2001 |
| [Group: Scleraxonians] |  |  |
| Family: Coralliidae Lamouroux, 1812 |  |  |
| *Corallium rubrum* Linnaeus, 1758 | AF413059 | Constantini et al., 2003 |
| *Corallium sp. 1* | GQ358526 | Herrera et al., 2010 |
| Family: Paragorgiidae Kükenthal, 1916 |  |  |
| *Paragorgia kaupeka* Sánchez, 2005 | GQ293292 | Herrera et al., 2010 |
| *Sibogagorgia cauliflora* Herrera, Baco & Sánchez, 2010 | GQ293288 | Herrera et al., 2010 |
| [Suborder: Holaxonians] |  |  |
| Family: Gorgoniidae Lamouroux, 1812 |  |  |
| *Africagorgia schoutedeni* Stiasny, 1939 | AY587533 | Aguilar & Sánchez, 2007a |
| *Gorgonia flabellum* Linnaeus, 1758 | AY587521 | Aguilar & Sánchez, 2007a |
| *Leptogorgia violacea* Pallas, 1766 | AY587527 | Aguilar & Sánchez, 2007a |
| *Lophogorgia* [Synonym of *Leptogorgia*] *euryale* Bayer, 1952 | AY587530 | Aguilar & Sánchez, 2007a |
| *Pacifigorgia stenobrochis* Valenciennes, 1846 | AY587531 | Aguilar & Sánchez, 2007a |
| *Pinnigorgia platysoma* Nutting, 1910 | AY587536 | Aguilar & Sánchez, 2007a |
| *Pseudopterogorgia* [Synonym of *Antillogorgia*] *bipinnata* Verrill, 1864 | AY587524 | Aguilar & Sánchez, 2007a |
| Family: Plexauridae Gray, 1859 |  |  |
| *Eunicea tourneforti* Milne Edwards & Haime, 1857 | EF490982 | Grajales et al., 2007 |
| *Muriceopsis bayeri* Sánchez, 2001 | AY587538 | Aguilar & Sánchez, 2007a |
| [Suborder: Calcaxonians] |  |  |
| Family: Isididae Lamouroux, 1812 |  |  |
| *Acanella weberi* Nutting, 1910 | FJ790943 | Dueñas & Sánchez, 2009 |
| *Acanella* sp. | FJ790921 | Dueñas & Sánchez, 2009 |
| *Isidella tentaculum* Etnoyer, 2008 | FJ790944 | Dueñas & Sánchez, 2009 |
| *Keratoisis zelandica* Grant, 1976 | FJ790939 | Dueñas & Sánchez, 2009 |
| *Lepidisis olapa* Muzik, 1978 | FJ790908 | Dueñas & Sánchez, 2009 |
| Family: Primnoidae Milne Edwards, 1857 |  |  |
| *Calyptrophora japonica* Gray, 1866 | EF090735 | Aguilar & Sánchez, 2007b |
|  |  |  |

**Table S5.** *Isis hippuris* ITS2 haplotype sequence view from the seven test sites within the WMNP. Each sequence represents haplotypes (in parentheses) present in each sample per site and GenBank accessions. Colour codes depict gaps (lilac), transitions (red), and transversions (yellow).

**Position/Site**

**Position/Site**


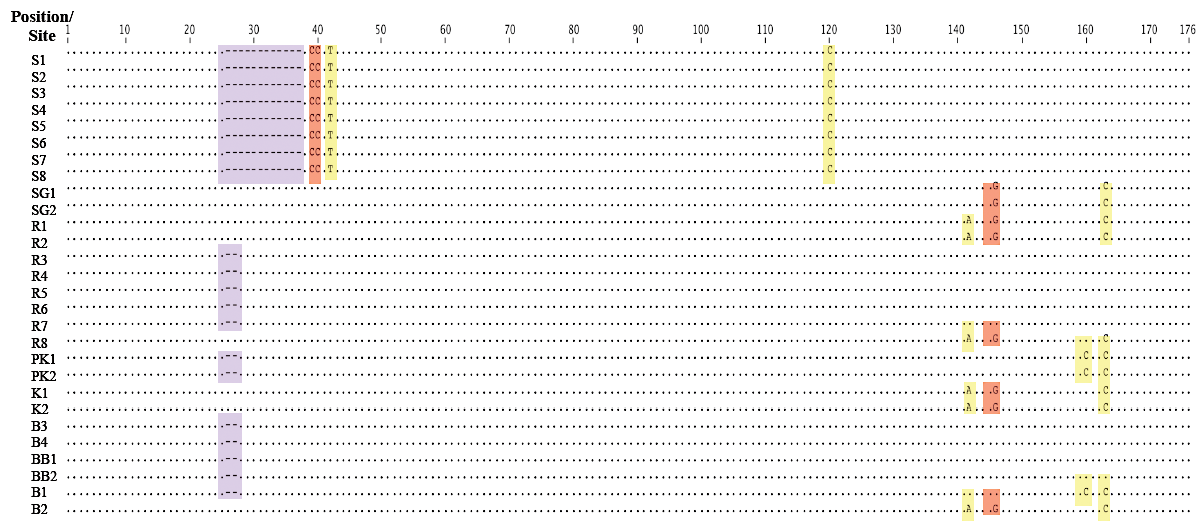


S1 (A) KP265677

S2 (A) KP265684

S3 (A) KP265683

S4 (A) KP265682

S5 (A) KP265681

S6 (A) KP265680

S7 (A) KP265679

S8 (A) KP265678

SG1 (D) KP265676

SG2 (D) KP265675

R1 (E) KP265690

R2 (E) KP265685

R3 (B) KP265694

R4 (B) KP265695

R5 (B) KP265696

R6 (B) KP265697

R7 (B) KP265698

R8 (E) KP265689

PK1 (C) KP265692

PK2 (C) KP265691

K1 (E) KP265688

K2 (E) KP265687

B3 (B) KP265699

B4 (B) KP265700

BB1 (B) KP265701

BB2 (B) KP265702

B1 (C) KP265693

B2 (E) KP265686

**Supplementary References**

**Aguilar C, Sánchez JA. 2007a.** Phylogenetic hypotheses of gorgoniid octocorals according to ITS2 and their predicted RNA secondary structures. *Molecular Phylogenetics and Evolution* **43**(3): 774-786.

**Aguilar C, Sánchez JA. 2007b.** Molecular morphometrics: contribution of ITS2 sequences and predicted RNA secondary structures to octocoral systematics. *Bulletin Marine Sciences* **81**(3): 335-349.

**Bayer FM. 1955.** Contributions to the nomenclature, systematics, and morphology of the Octocorallia. *Proceedings of the United States National Museum* **105**(3357): 207-220, pl.8.

**Bayer FM, Stefani J. 1987.** Isididae (Gorgonacea) de Nouvelle-Calédonie--Nouvelle clé des genres de la famille. *Bulletin of the Museum of Natural History Nature Paris*, (4 sér.) **9** (section A) No. 1:47-106, pls. 1-30.

**Constantini F, Tinti F, Abbiati M. 2003.** Sistematica molecolare e filogenesi di *Corallium rubrum*. *Biologia Marina Mediterranea* **10**: 73–75.

**Dueñas LF, Sánchez JA. 2009.** Character lability in deep-sea bamboo corals (Octocorallia, Isididae, Keratoisidinae). *Marine Ecology Progress Series* **397**: 11-23.

**Fabricius KE, Alderslade P. 2001.** *Soft corals and sea fans: a comprehensive guide to the tropical shallow-water general of the Central-West Pacific, the Indian Ocean and the Red Sea*. AIMS (AIDAB), Townsville. pp. 264.

**Grajales A, Aguilar C, Sánchez JA. 2007.** Phylogenetic reconstruction using secondary structures of Internal Transcribed Spacer 2 (ITS2, rDNA): finding the molecular and morphological gap in Caribbean gorgonian corals. *BMC Evolutionary Biology* **7**: 90.

**Herrera S, Baco A, Sánchez JA. 2010.** Molecular systematics of the bubblegum coral genera (Paragorgiidae, Octocorallia) and description of a new deep-sea species. *Molecular Phylogenetics and Evolution* **55**(1): 123-135.

**Kölliker RA. 1865.** *Die Bindesubstanz der Coelenteraten*. Icones histologicae oder Atlas der vergleichenden Gewebelehre. Leipzig, Germany, pp. 87-181.

**Kükenthal W. 1915.** Das System der Seefedern. *Zoologischer Anzeiger* **45**(6): 284-287.

**Kükenthal W. 1919.** *Gorgonaria.* Wissenschaft. Ergebn. Deutsch. Tiefsee-Exspedition auf dem Dampfer Valdivia 1898–1899, Band 13, 946 pp.

**Kükenthal W. 1924.** *Coelenterata: Gorgonaria*. Das Tierreich 47. Berlin: Walter de Gruyter and Co. pp.478.

**Linnaeus C. 1758.** Systema naturae per regna tria naturae :secundum classes, ordines, genera, species, cum characteribus, differentiis, synonymis, locis (in Latin) (10th ed.). Stockholm: Laurentius Salvius.

**Mai-Bao-Thu F, Domantay JS. 1971.** Taxonomic studies of the Philippine gorgonaceans in the collections of the University of Santo Tomas, Manila (cont’d). *Acta Manilana* **7**: 3-77.

**McFadden CS, Donahue R, Hadland BK, Weston R. 2001.** A molecular phylogenetic analysis of reproductive trait evolution in the soft coral genus *Alcyonium*. *Evolution* **55**: 54-67.

**Milne-Edwards H, Haime J. 1857.** *Histoire naturelle des coralliaires, ou polypes proprement dits*. Paris, Roret. pp. 326.

**Nutting CC. 1910.** *The Gorgonacea of the Siboga Expedition V. The Isidae*. Siboga- Expeditie Monograph, 13b2, 1–24.

**Rowley SJ. 2014.** Gorgonian responses to environmental change on coral reefs in SE Sulawesi, Indonesia. Doctoral thesis, Victoria University Wellington, New Zealand, pp. 213.

**Simpson JJ. 1906.** The structure of *Isis hippuris*, Linnaeus. *Journal of the Linnean Society of London, Zoology* **29**(194): 421-434.

**Stiasny G. 1940.** *Biological results of the Snellius Expedition*. VII. Die Gorgonarien Sammlung.

**Thomson JA, Simpson JJ. 1909.** An account of the alcyonarians collected by the Royal Indian Marine Survey Ship Investigator in the Indian Ocean; with a report on the species of *Dendronephthya* by Henderson WD II. The alcyonarians of the littoral area. The Indian Museum, Calcutta.

**Wright EP, Studer TH. 1889.** Report on the Alcyonaria. Rep. Scient. Results Explor. *Voyage Challenger.* **31**(1): 1-314.

**Zou R, Huang B, Wang X. 1991.** Studies on the gorgonians of China – I. *Isis* with one new species. *Acta Oceanologica Sinica* **10**(4): 593-602.
